# Predicting COVID-19 and Influenza Vaccination Confidence and Uptake in the United States

**DOI:** 10.3390/vaccines11101597

**Published:** 2023-10-15

**Authors:** Lijiang Shen, Daniel Lee

**Affiliations:** Department of Communication Arts and Sciences, Pennsylvania State University, University Park, PA 16802, USA; dal5666@psu.edu

**Keywords:** vaccination, COVID-19, influenza, reasoned action, integrated behavioral model, Tobit regression

## Abstract

This study investigates and compares the predictors of COVID-19 and influenza vaccination confidence and uptake in the U.S. Vaccine hesitancy is defined as the reluctance or refusal (i.e., less than 100% behavioral intention) to vaccinate despite the availability of effective and safe vaccines. Vaccine hesitancy is a major obstacle in the fight against infectious diseases such as COVID-19 and influenza. Predictors of vaccination intention are identified using the reasoned action approach and the integrated behavioral model. Data from two national samples (*N* = 1131 for COVID-19 and *N* = 1126 for influenza) were collected from U.S. Qualtrics panels. Tobit regression models were estimated to predict percentage increases in vaccination intention (i.e., confidence) and the probability of vaccination uptake (i.e., intention reaching 100%). The results provided evidence for the reasoned approach and the IBM model and showed that the predictors followed different patterns for COVID-19 and influenza. The implications for intervention strategies and message designs were discussed.

## 1. Introduction

### 1.1. Predicting COVID-19 and Influenza Vaccination Confidence and Uptake

COVID-19 (SARS-CoV-2) and the flu (influenza) are two contagious viruses that have spread throughout the world. The World Health Organization (WHO) classified COVID-19 as a pandemic in March 2020 because of its high transmission rates and high estimated fatality rates. Additionally, the flu can similarly create life-threatening illnesses in people who are infected. Both COVID-19 and influenza viruses are most often spread when a person comes into contact with droplets or aerosols that contain the virus (from an infected person). During the COVID-19 pandemic, due to the similarities in their symptoms, the exclusion of the flu could expedite the diagnosis of COVID-19. In both cases, vaccines are the most effective way to prevent the virus from spreading, hospitalization, or death. 

Vaccine hesitancy is a barrier to vaccination efforts that is frequently investigated by health communication scholars and practitioners [1]. Vaccine hesitancy describes those cases where individuals have access to the vaccine but have not received the vaccine by choice; it is one of the 10 most substantial threats to human health according to the WHO, in part because lower vaccination rates allow viruses to continue spreading via novel variants. For COVID-19, a vaccination rate of around 70–85% of a given population creates herd immunity [2], such that the virus does not have enough hosts in a given population to continue evolving into novel strains, while a vaccination rate of about 83% creates herd immunity for the flu.

A recent meta-analysis of over 40 studies on COVID-19 vaccines found that only about 61% of all participants across studies were accepting of the COVID-19 vaccines [3], and another study found that only 61.6% of participants intended to receive a flu vaccine [4]; both were well below the level needed for herd immunity. This indicates that herd immunity seems a distant and almost impossible goal for either virus due to vaccine hesitancy. While the COVID-19 virus pandemic formally ended in May of 2023, the disease is still capable of spreading and causing harm. Meanwhile, influenza spreads throughout the world annually. Given the prevalence of these viruses amidst insufficient rates of vaccination, understanding predictors of both vaccine hesitancy and intentions to vaccinate could provide implications and guidance for intervention strategy designs; hence, it is crucial to efforts to increase vaccination rates and protect individuals from these viruses.

Such is the goal of this study. Within the framework of the reasoned action approach [5] and the integrated behavioral model (IBM; [6]), predictors of vaccination intention will be identified. Data collected from two U.S. national samples recruited from Qualtrics panels will be analyzed to test the hypotheses and answer the research questions. The differences and similarities between COVID-19 and influenza will be analyzed. Implications for vaccine hesitancy and message strategies to enhance vaccine confidence/uptake will be discussed.

### 1.2. Vaccine Hesitancy

Vaccine hesitancy has been one of the most dominant issues during the COVID-19 pandemic. Vaccine hesitancy is formally defined by the WHO as the reluctance or refusal to vaccinate despite the availability of vaccines. This highlights a behavioral approach to defining the construct (see [7] for a review of definitions of vaccine hesitancy). Vaccine-related behaviors can range from accepting vaccines with no doubts, to complete refusal with no doubts, with heterogeneous vaccine hesitancy groups between the two poles [8]. Individuals who have already received or are 100% ready and committed to receiving the vaccine upon availability and/or eligibility are considered vaccine-inclined or -confident (but see implementation intention [9]. Vaccine-hesitant individuals include those who refuse vaccines and those who delay the acceptance of vaccines despite their availability, effectiveness, and safety [10,11]. Any individual who has not received the vaccine; is less than 100% ready; or has not yet decided to receive it (at a measurement moment), is considered vaccine-hesitant.

Given such an approach to vaccine hesitancy, the reasoned action approach [5] and the IBM model [6] offer clear guidance regarding potential antecedents of behavioral intention in general, and intention to receive a vaccine specifically. The IBM model postulates the following antecedents to behavioral intention: (a) affect (i.e., feelings and emotions) and behavioral beliefs, which determine (experiential and instrumental) attitude, (b) (descriptive and injunctive) normative beliefs, and control and efficacy beliefs. The IBM model also includes such factors as knowledge and skills, salience of behavior, environmental constraints, and habit. But these factors are believed to influence behavior directly, rather than via behavioral intention. Background factors such as demographic variables, socio-economic variables, influences from the media/social media, and individual differences variables are assumed to exert their influence on intention through the above-mentioned antecedents in varying ways. There is robust evidence for the utility of the model in understanding and predicting health behaviors [5] (see also [12,13,14]).

### 1.3. Predictors of Vaccination Intention

#### 1.3.1. Attitude toward Vaccines and Its Antecedents

The reasoned action approach to behavior [5] posits that behavioral intention is a direct function of attitude. The attitude–behavior link is well-established in the literature (see [15]). The integrated behavioral model argues that antecedents to attitude include both affect and cognition (e.g., beliefs and evaluations). 

**Affect**. Vaccine-induced or -related emotions contribute to vaccine hesitancy and declines in vaccine uptake [10,16,17]. Anti-vaccination groups have long been using emotions as strategies in promoting misinformation and conspiracy theories, which may have a more powerful influence than didactic scientific statements on probabilities [10,16]. Individuals might have quite different affective responses to COVID-19 vs. influenza and their vaccines. The fact that COVID-19 vaccines had gone through an unusually rapid development process and the new technology (mRNA) used in their development sparked new fears over vaccine safety [10]. Sadness occurs in response to the loss of lives and COVID-related pain and suffering in general. With the politicization of the COVID-19 situation, safety measures including mask mandates and lockdowns were controversial and aroused anger and disgust at disagreeable advocacies from polarized groups. On the other hand, effective and safe vaccines offer hope for the end of the COVID-19 pandemic and the return to normalcy, which can elicit other positive emotions such as happiness and calmness. There has been empirical evidence for the impact of affect on vaccine hesitancy [18,19,20,21].

There are two approaches to the impact of affect on attitude: the dual-dimensional approach and the discrete emotions approach [22]. The dual-dimensional approach to affect posits that affective experiences arise from two fundamental neurophysiological systems, one related to valence (a pleasure–displeasure continuum) and the other to arousal (an activation–deactivation continuum). Each affect is determined by a linear combination of these two dimensions [23]. The dual-dimensional approach suggests that the impact of affect on attitude is rooted in its valence. This is consistent with affect as an information perspective [24,25]. Given that affect results from an individual’s assessment of the person–environment relationship, negative affect arises from the perception that the environment or the object under evaluation is harmful or at least incongruent with the individual’s goals. On the other hand, positive affect takes place when the environment or object under evaluation is deemed to be beneficial and conducive to the individual’s goals (see [17]). Hence, it was predicted that:

**H1a.**  *The valence of individuals’ vaccine-induced emotions is positively correlated with their attitude toward vaccines.*

The discrete emotions approach shares the assumption that affect arises from the evaluation of the person–environment relationship but posits that the structure of affect is more complicated. A family of theories concur that emotions are patterned changes in multiple biopsychosocial systems [26] including (a) cognitive processes, (b) subjective experience, (c) expression, (d) physiology, (e) motivation, and (f) neural correlates ([27]. Discrete emotions are those feelings that manifest distinctive patterns of changes across these various systems. Each specific emotion is determined by different patterns of cognitive appraisals [28]. This approach suggests that the impact of affect on attitude is a function of the signal value, function, and action tendency of emotions [22]. Hence it was predicted that:

**H1b.**  *Similarly-valenced vaccine-induced emotions are associated with attitude toward vaccines in different patterns in terms of direction and magnitude*.

**Behavioral Beliefs.** Consistent with the cognitive response theories, the reasoned action approach proposes that behavioral beliefs or cognitive appraisals of vaccine-related issues (e.g., the threat from the viruses, the vaccines, etc.) are determinants of attitude toward vaccines [5] (see also the protection motivation theory, [29]). Specifically, (a) threat appraisals of the viruses (severity and susceptibility) and (b) appraisals of vaccine potency (effectiveness and safety) as a solution to reduce/prevent the danger from the viruses would lead to positive attitudes toward viruses (as a solution to reduce/prevent the danger). On the other hand, the perceived risk of vaccine-related side effects (severity and susceptibility) would result in negative attitudes toward vaccines [30]. Evidence for the impact of cognitive factors on attitude has been well-documented in meta-analyses (e.g., [12,13,31]. Therefore, it was predicted that:

**H2a.**  *Perceived virus severity and susceptibility are positively associated with attitude toward vaccines.*

**H2b.**  *Perceived effectiveness of vaccines is positively associated with attitude toward vaccines.*

**H2c.**  *Perceived severity of and susceptibility to vaccine side effects are negatively associated with attitude toward vaccines.*

**Misinformation Beliefs**. Misinformation can be broadly defined as information whose accuracy is not or cannot be verified and is spread through various channels, such as social media, news outlets, and word of mouth, with or without the intention to cause harm [32]. The past three decades have witnessed increasing concerns over the risks of vaccines, particularly the alleged consequences of triggering autism in children [33]. Misinformation was particularly rampant during the COVID-19 pandemic [34]. The conspiracy theory video *Plandemic* emerged long before the COVID-19 vaccines were available. The video was quickly taken down from social media sites, but its impact lingers—belief in misinformation is the leading predictor of vaccine hesitancy [17,35,36,37,38,39,40]. The content and nature of the misinformation are usually connected to outcomes or consequences of vaccines, which impacts attitude directly according to the reasoned action approach. Hence, it was predicted that:

**H3.**  *Vaccine-related misinformation beliefs are negatively associated with attitudes toward vaccines.*

The affective and cognitive antecedents to attitude toward vaccines and the robust attitude–behavior relationship [15] mean that these factors have significant indirect effects on vaccination intention. However, the reasoned action approach [5] does not explicitly state if attitude completely mediates the impact of these factors on intention. Meanwhile, other models suggest there might be direct effects from cognitive appraisal variables on intention, that is, above and beyond their impact mediated by attitude (e.g., protection motivation theory [29]; health belief model, [41]). Hence, a research question was asked:

**RQ1**: Does attitude completely mediate the impact of affect, behavioral beliefs, and misinformation beliefs on vaccination intention?

#### 1.3.2. Social Norms

Social norms are the second type of factor that predicts behavior intention [5,42]. There are two types of norms, descriptive and injunctive. Descriptive norms refer to the perceived behavior by the majority or a typical person. Descriptive norms tend to serve the epistemic function of reducing uncertainty when a person is new to an environment or social context [43].Compliance with descriptive norms is driven by the desire to fit in, while noncompliance might not involve social sanctions [44]. Injunctive norms refer to the belief of what ought to be done or perceived social rules that individuals have to follow based on important others’ opinions. Compliance with injunctive norms is motivated by the need for overt social recognition, and norm violations oftentimes result in disapproval or punishment [45,46]. The evidence for normative influence on behavior is also well-documented in meta-analyses [12,31,47,48,49]. Hence, it was predicted:

**H4.**  *Descriptive and injunctive norms are positively associated with vaccination intention*.

In the context of COVID-19 vaccination, which is a personal and private behavior, both descriptive norms and injunctive norms have been found to predict vaccination intention, but the impact of injunctive norms was weaker than that of descriptive norms [50,51], although their effect might not be as robust [52]. The differences between COVID-19 and influenza and their vaccines [53] might add more uncertainty: The two vaccines differ from each other substantially when it comes to novelty, the technology used to develop the vaccines, the uncertainty involved regarding vaccine effectiveness, safety, and potential side effects. In other words, the situation and context of COVID-19 vaccines are more novel and more uncertain than those of influenza vaccines, which indicates that normative norms should be more influential than injunctive norms for COVID-19 vaccination intentions (more so than for influenza vaccination intentions). On the other hand, the COVID-19 vaccine has been a very politically charged and deeply divisive issue: on one side, there were calls for herd immunity [2] and people advocating for vaccine mandates, and on the other side, people have been openly protesting against and resisting the vaccines, which suggests there are conflicting injunctive norms. There is no clear guidance from theory or empirical evidence regarding the differential effects on vaccination intention between COVID-19 and influenza vaccines. Along this line of argument, the following was advanced: 

**RQ2**: Do descriptive and injunctive norms have differential effects on vaccination intention for COVID-19 vs. influenza?

#### 1.3.3. Efficacy and Control Beliefs

The final category of factors argued to directly influence intentions to perform a behavior is individuals’ perception of personal agency, or their perceived capacity to perform some action ([5,6,54]). Perceived control describes the extent to which individuals believe that environmental conditions promote, or inhibit, their ability to perform a target behavior, while self-efficacy describes the extent to which individuals feel confident that they can overcome obstacles that inhibit perceived control in order to perform a given behavior. Empirical evidence for the impact of control beliefs and self-efficacy on behavioral intention is also well-documented [12,31]. Specifically in the context of COVID-19 vaccines, there is also evidence for the impact of self-efficacy on vaccination intention [19,20]. Therefore, it was predicted:

**H5.**  *Self-efficacy and vaccination intention are positively correlated.*

#### 1.3.4. Previous Vaccination Behavior

Individuals have the general tendency to repeat their behaviors. On one hand, they desire consistency and momentum in their behavior [55] (see also the flexible stability of attitude, [56]). On the other hand, the same personality and motivational factors might be at play in similar situations across time, and past behavior may serve as a heuristic cue that guides decision-making and future actions [57], as well as exerting influences on behavior through cognitive and affective antecedents [6,58,59,60]. The evidence for the role of past behavior in influencing behavioral intention and future behavior has been well-documented in meta-analyses as well [12,61]. Along this line of argument, it was predicted that,

**H6.**  *Previous vaccination behavior is positively associated with vaccination intention.*

### 1.4. The Difference between COVID-19 and Influenza Vaccines

While the pattern regarding the antecedents and correlates of vaccination intention is expected to hold for both COVID-19 and influenza, there are also potential differences since vaccine hesitancy is context-specific and could vary across vaccines [8]. Researchers have compared the COVID-19 vaccine to the case of influenza [53] in the sense that we need to learn to live with both viruses and that booster shots and annual revaccination might be required to avert severe consequences. On the other hand, the two topics also differ from each other substantially when it comes to novelty, the technology used to develop the vaccines, the uncertainty involved regarding vaccine effectiveness, safety, and potential side effects, and how individuals react to the disease as well as to the vaccines. The introduction of new vaccines (e.g., COVID-19 vaccines), in particular, oftentimes encounters higher hesitancy due to the relatively higher levels of uncertainty involved. They are also easy targets for anti-vax individuals and groups. Given these similarities and differences, the antecedents might potentially have a differential impact on vaccination intention, although the overall pattern should hold between the two topics. Hence, it was asked,

**RQ3**: Do the antecedents have a differential impact on vaccination intention between COVID-19 and influenza vaccines?

## 2. Method

### 2.1. Participants and Procedure

Data collection took place in December 2022. Participants were sampled from the general U.S. population recruited through a national paid opt-in online panel comprised of individuals who registered with Qualtrics. Table 1 presents the demographic information of the sample. Consenting participants responded to a set of demographic questions, followed by their vaccination history (COVID-19 and influenza), risk perception related to COVID-19 and influenza, and political orientation before they were randomly assigned to either the topic of COVID-19 or influenza vaccine. The questions within each topic assessed cognitive and affective responses to vaccines, perceived vaccine effectiveness and safety, perceived risks of vaccine side effects, and descriptive and injunctive norms related to the vaccine. Toward the end of the questionnaire, participants reported their attitude toward, and intention to receive annual COVID-19 re-vaccination (if recommended) or the influenza vaccine.

To improve data quality, respondents who failed at attention-checker questions were automatically dropped from the sample. Respondents whose participation lasted less than 1/3 of the mean duration in the soft launch (*M* = 13.5 min) were also automatically dropped. Data were also carefully examined for patterns of straight-lining and these cases were dropped when detected.

### 2.2. Measures

The unidimensionality of multi-item scales was established with confirmatory factor analysis (CFA). All items were measured on a 7-point Likert scale ranging from “1 = Strongly disagree” to “7 = Strongly agree” unless stated otherwise.

#### 2.2.1. Vaccine-Induced Emotions

This set of variables was assessed by asking participants to indicate the extent to which they had the following feelings about COVID-19/influenza vaccines on a 7-point scale (0 = *none of this feeling*, 6 = *a lot of this feeling*) adapted from [62]. The emotions and their items (with reliabilities for COVID-19 and influenza) were: hope (hopeful, optimistic, upbeat, α = 0.82, 0.87), happiness (happy, glad, delighted, joyful, α = 0.92, 0.92), sadness (sad, depressed, dismal, dreary, α = 0.88, 0.89), fear (scared, afraid, fearful, α = 0.92, 0.89), anger (annoyed, irritated, angry, α = 0.89, 0.89), disgust (disgusted, grossed out, repulsed, α = 0.86, 0.89), worry (worried, anxious, nervous, α = 0.89, 0.88), and guilt (guilty, ashamed, embarrassed, α = 0.89, 0.88).

#### 2.2.2. Perceived Virus Severity

Perceived severity of COVID-19 and influenza was measured by four items: “COVID-19/the flu is harmful”, “dangerous”, “COVID-19/the flu is a serious problem nationally”, and “in my local community” (α = 0.86 for COVID-19 and 0.81 for influenza). 

#### 2.2.3. Perceived Virus Susceptibility 

Perceived susceptibility to COVID-19/influenza was assessed with four items: “There is a chance that I/someone I cared about could contract COVID-19” and “It’s possible/there is a chance that I/someone I cared about could get COVID-19/the flu” (α = 0.86 for COVID-19 and 0.88 for influenza).

#### 2.2.4. Perceived Vaccine Effectiveness

Perceived COVID-19 and influenza vaccine effectiveness were assessed with three items: “the COVID-19 vaccines/flu shot is effective/work(s) in preventing COVID-19/the flu and its complications,” and “If I get the vaccine, I will be less likely to get COVID-19/the flu” (α = 0.95 for COVID-19 and 0.92 for influenza).

#### 2.2.5. Perceived Severity of Vaccine Side Effects

Perceived severity of vaccine side effects was measured by three items: “The COVID-19 vaccine/flu shot could have serious/harmful/dangerous side effects” (α = 0.95).

#### 2.2.6. Perceived Susceptibility to Vaccine Side Effects

Perceived susceptibility to vaccine side effects was assessed by three items: “I could be affected by the side effects of COVID-19 vaccines/flu shot”, and “There is a chance/It is likely that I might be affected by the side effects of COVID-19 vaccines/flu shot” (α = 0.94 for COVID-19 and 0.92 for influenza).

#### 2.2.7. Vaccine-Related Misinformation Beliefs

Belief in vaccine-related misinformation was assessed with different scales for the two topics. For COVID-19 vaccines, the items were: “COVID-19 vaccines were rushed and wouldn’t be effective”, “The mRNA COVID-19 vaccines could alter your DNA”, “The COVID-19 vaccines could lead to infertility”, “The COVID-19 vaccines make your magnetic”, “The COVID-19 vaccines were made from fetal tissue”, “The COVID-19 vaccines have a microchip to track you”, and “The COVID-19 vaccines could have long-term health complications”. The items for influenza were: “The flu vaccine can give you the flu”, “If a person has a chronic illness or is pregnant, they shouldn’t get the flu vaccine”, “People don’t need the flu vaccine every year”, “The flu vaccines were rushed and wouldn’t be effective”, “People who get the flu vaccine still get the flu, so it’s not worth it”, and “If people don’t get the flu vaccine early in the season, it’s too late now”. Belief in vaccine-related misinformation is considered a formative measure whose indicators cause the latent variable; hence alpha reliability is irrelevant [63].

#### 2.2.8. Attitude toward Vaccines

Attitude toward the COVID-19/influenza vaccines was assessed with six 7-point semantic differential items. The word pairs were: unimportant/important, bad/good, negative/positive, unwise/wise, threatening/assuring, and risky/safe (α = 0.96 for COVID-19 and 0.95 for influenza).

#### 2.2.9. Descriptive Norms

Vaccine-related descriptive norms were measured by four items: “Most people I know/Most of my family and loved ones/Most people I work/go to school with/Most people in my community *have received* the COVID-19/influenza vaccines” (α = 0.88 for COVID-19 and 0.89 for influenza).

#### 2.2.10. Injunctive Norms

Vaccine-related injunctive norms were measured by four items: “Most people I know/Most of my family and loved ones/Most people I work/go to school with/Most people in my community *think I should* get the COVID-19/influenza vaccines” (α = 0.92 for COVID-19 and 0.93 for influenza).

#### 2.2.11. Perceived Self-Efficacy to Receive Vaccines

Perceived self-efficacy to receive the COVID-19/influenza vaccines was assessed by three items: “If I want to, I am confident I could get the COVID-19 booster/flu shot”, “I have the ability to get the COVID-19 booster/flu shot”, and “Getting the COVID-19 booster/flu shot would be difficult for me” (reverse coded) (α = 0.78 for COVID-19 and 0.76 for influenza).

#### 2.2.12. Previous Vaccination Behavior

For COVID-19 vaccination behavior, participants were asked to report if they have received the updated COVID-19 booster shot, the first booster shot, the primary series (i.e., two doses of the Pfizer or Moderna vaccines or a single shot of the Johnson & Johnson vaccine), or no vaccines. For influenza vaccination behavior, participants were asked to report if they had received the influenza vaccine for the 2022–2023 flu season.

#### 2.2.13. Vaccination Intention

On a 0–100 scale (0% = *not likely at all*, 100% = *absolutely*), participants indicated on a slider either their likelihood (in percentage) of getting annual re-vaccination for COVID-19 if it is recommended by the CDC [64] or the influenza vaccine for the current flu season (if they have not received the vaccine yet) or the next flu season (if they have received the vaccine for this year).

#### 2.2.14. Political Orientation

The vaccine hesitancy literature also suggests significant associations between political orientation and vaccine hesitancy; that is, more conservative individuals tend to be more vaccine-hesitant than more liberal individuals [65,66,67], although not all conservatives are vaccine-hesitant [39]. Political orientation was measured by two 1–7-point semantic differential items. The word pairs were: liberal/conservative and left-wing/right-wing. A composite score was created by taking the average of the two items (*r* = 0.86), where higher scores indicate a stronger conservative orientation.

## 3. Results

### 3.1. Preliminary Results

This study is part of a larger project. Other parts of the data were reported in Shen (2023). The data files used in this study are available at: https://osf.io/4y3h6/?view_only=c89dfbff34c84731bcbaf932fa726b51.

Among the COVID-19 sample, *n* = 273, or 24.1% were unvaccinated (i.e., received zero shots, or only one shot of the Pfizer or Moderna vaccine); *n* = 334, or 29.6% completed the primary series; *n* = 386, or 34.1% received a booster shot; and *n* = 138, or 12.2% received the updated booster shot. For vaccination intention, *n* = 324, or 28.6% reported that it was 100% that they would receive an annual COVID-19 shot (if it is recommended). Among the influenza sample, *n* = 600, or 53.3% received the influenza vaccine, and *n* = 526, or 46.7%, did not. For vaccination intention, *n* = 414, or 37.8% reported it was 100% that they would receive the influenza vaccine for the current (if they have not received the vaccine this year) or the next flu season (if they have received the vaccine this year).

### 3.2. Data Analysis Strategy

The preliminary results showed that there were a large number of participants who reported it was 100% that they would receive either an annual COVID-19 shot (if recommended) (28.6%) or the influenza vaccine (37.8%). This means the two vaccination intention variables had *censored* distributions (Censored distributions can occur when values exist outside of a measuring instrument. For example, when the range of a clinical thermometer is between 35 and 39 degrees Celsius, values below 35 degrees will be recorded as 35 and values above 39 degrees as 39, resulting in a censored distribution. Censored distributions can also occur because of the nature of the phenomenon studied (i.e., due to ceiling or flooring effects), as is the case here—there are no values outside of the range of 0–100% intention. It just so occurred that substantial proportions of the sample were clustered at the upper threshold of 100%), where considerable proportions of observations are clustered at a (lower and/or upper) threshold (100% and upper threshold in this case), while a substantial amount of variances remain above or below the threshold (see [68,69] for more discussions on censored distributions). *Truncated* distributions are similar to censored distributions in that truncated distributions also have a restricted/limited range but have a density proportional to a normal distribution (i.e., there is no substantial number of cases clustered at the threshold). In this study, there was a substantial amount of variance for the proportion of data below the threshold of 100% (*M* = 44.10%, *SD* = 36.64 for COVID-19; *M* = 40.26% and *SD* = 36.30 for influenza). Such censored distributions mean that estimates from linear models may be biased in the form of underestimation [70]. In this case, the Tobit procedure [69,71,72] is more appropriate and yields consistent and robust parameter estimates. 

There are also advantages to the Tobit procedure from the substantive and conceptual perspectives (see also [73,74]). The ultimate goal in interventions is vaccine uptake. Such concrete and tangible behavioral changes might be difficult and gradual [75]. The increase in intention might be a prerequisite (but see [58]). For the purpose of promoting vaccine uptake and building vaccine confidence, it is important to understand both (a) what drives individuals to be 100% ready for the vaccines (i.e., becoming vaccine-confident/-inclined), and (b) what increases the likelihood of vaccination even if it does not reach 100% (i.e., building vaccine confidence and reducing vaccine hesitancy). These two types of changes are both qualitatively and quantitatively different. Correspondingly, the Tobit procedure simultaneously estimates (a) a marginal effect, which refers to the impact of each predictor on the probability of an individual becoming vaccine-confident/-inclined (i.e., one’s vaccination intention reaching the threshold of 100%), and (b) a linear effect, which is the impact of each predictor on the individual’s vaccination intention (i.e., likelihood in percentages) given that it remains below the threshold of 100%. Coefficient estimates from Tobit models have to be decomposed into these two effects for substantive interpretations. Therefore, the strategy of the Tobit model was adopted for data analysis when predicting vaccination intention. The ordinary least squares (OLS) regression approach was adopted otherwise.

### 3.3. Statistical Power

Power analysis was performed with G*Power [76]. At the level of α < 0.05, six covariates (demographic variables and political orientation) and 16 tested predictors, an effect size equivalent to Cohen’s *f*^2^ = 0.10, the sample sizes of *N* = 1131 (COVID-19) and *N* = 1126 (influenza) yielded a statistical power exceeding 0.99.

### 3.4. Hypotheses and Research Questions

The first three hypotheses did not involve vaccination intention, but attitude. OLS regression models were estimated within each topic to predict attitude toward vaccines. In each model, the demographic variables were entered in the first block, and previous vaccination behavior, religiosity, and political orientation were entered in the second block. The emotion variables were entered in the third block. Finally, the cognitive appraisals and misinformation beliefs were entered in the fourth block. Table 2 presents the OLS results.

H1a and H1b are concerned with the impact of vaccine-induced emotions on attitude. The emotions that significantly predicted attitude toward COVID-19 vaccines were worry (β = −0.07, *p =* 0.03) and guilt (β = 0.09, *p <* 0.001). Anger (β = −0.05, *p =* 0.06) and disgust (β = −0.05, *p =* 0.06) were significant predictors at α < 0.10. Neither of the two positive emotions (hope and happiness) was a significant predictor. The emotions that significantly predicted attitude toward influenza vaccines were happiness (β = 0.09, *p =* 0.02), worry (β = −0.12, *p =* 0.001), disgust (β = −0.07, *p =* 0.02), and guilt (β = 0.14, *p <* 0.001). Hope (β = 0.07, *p =* 0.06) and sadness (β = 0.07, *p =* 0.08) were significant predictors at α < 0.10. These results showed some support for H1a in that the effects of the positive emotions (happiness and hope) on attitude toward influenza vaccines were in line with their valence across the two topics. The results also showed support for H1b in that the negative emotions predicted attitude in different directions: The effects of guilt and sadness were positive, and the effects of worry, disgust, and anger were negative. This was consistent with previous findings [22] that the role of affect in persuasion is best explained by the dual-dimensional and discrete emotion approaches combined.

H2 and H3 were about the impact of the cognitive factors. The patterns were consistent across the two vaccines: Perceived virus severity (β = 0.10, *p <* 0.001 for COVID-19 and β = 0.07, *p =* 0.001 for influenza), perceived vaccine effectiveness (β = 0.44, *p <* 0.001 for COVID-19 and β = 0.33, *p <* 0.001 for influenza), and perceived side effects severity (β = −0.12, *p <* 0.001 for both COVID-19 and influenza) were significant predictors, but not perceived susceptibility to the viruses or vaccine side effects. H2a and H2c received partial support, and H2b received support. Misinformation beliefs were negatively associated with attitude toward vaccines for both COVID-19 (β = −0.12, *p <* 0.001) and influenza (β = −0.17, *p <* 0.001). H3 received support.

H4–6 and the RQs concerned with vaccination intention. A series of Tobit models were estimated for COVID-19 and influenza, respectively, to predict vaccination intention. To answer RQ1, two models were estimated: The demographic variables, political orientation, previous vaccination behavior, and the affective and cognitive antecedents of attitude were entered as predictors in Model 1. Attitude was entered as an additional predictor in Model 2.

For COVID-19, happiness (*b* = 1.81, *p =* 0.04), perceived virus severity (*b =* 4.82, *p <* 0.001), vaccine effectiveness (*b =* 9.16, *p <* 0.001), perceived side effects severity (*b =* −4.54, *p <* 0.001), and misinformation beliefs (*b =* −2.36, *p =* 0.009) were significant predictors in Model 1. Model 2 explained a significant amount of variance above and beyond Model 1 (∆pseudo $R^{2}$ = 0.007, *p <* 0.001). Attitude was a significant predictor (*b =* 7.19, *p <* 0.001) in Model 2. Perceived virus severity (*b =* 3.91, *p <* 0.001), perceived vaccine effectiveness (*b =* 6.11, *p <* 0.001), and perceived side effects severity (*b =* −3.53, *p <* 0.001) remained significant predictors. Happiness (*b =* 1.53, *p =* 0.08) and misinformation beliefs (*b =* −1.33, *p =* 0.13) were no longer significant predictors. 

For influenza, hope (*b =* 3.89, *p =* 0.001), happiness (*b =* −2.74, *p =* 0.02), worry (*b =* −3.92, *p =* 0.002), perceived virus severity (*b =* 2.80, *p =* 0.002), perceived virus susceptibility (*b =* 3.15, *p =* 0.001), perceived vaccine effectiveness (*b =* 8.21, *p <* 0.001), perceived side effects susceptibility (*b =* −2.57, *p =* 0.01), and misinformation beliefs (*b =* −6.24, *p <* 0.001) were significant predictors in Model 1. Model 2 explained a significant amount of variance above and beyond Model 1 (∆pseudo $R^{2}$= 0.007, *p <* 0.001). In Model 2, attitude was a significant predictor (*b =* 7.64, *p <* 0.001). Hope (*b =* 3.26, *p =* 0.003), happiness (*b =* −3.17, *p =* 0.004), worry (*b =* −2.88, *p =* 0.02), perceived virus severity (*b =* 2.16, *p =* 0.01), perceived virus susceptibility (*b =* 2.99, *p =* 0.001), perceived vaccine effectiveness (*b =* 5.51, *p <* 0.001), perceived side effects susceptibility (*b =* −2.57, *p =* 0.007), and misinformation beliefs (*b =* −4.49, *p <* 0.001) remained significant predictors. These results showed that attitude toward vaccines did not completely mediate the impact of its affective and cognitive antecedents on vaccination intention (RQ1).

To test H4–H6 and answer RQ2 and RQ3, descriptive and injunctive norms and self-efficacy were entered in Model 3. For COVID-19, Model 3 explained additional variance compared to Model 2 (∆pseudo $R^{2}$ = 0.003, *p <* 0.001). Descriptive norms (*b =* −4.85 *p <* 0.001), injunctive norms (*b =* 5.85, *p <* 0.001), self-efficacy (*b =* 1.54, *p =* 0.04), and previous vaccination behavior (*b =* 11.17, *p <* 0.001) were significant predictors. For influenza, Model 3 also explained additional variance compared to Model 2 (∆pseudo $R^{2}$ = 0.005, *p <* 0.001). Descriptive norms (*b =* 3.81, *p =* 0.007), injunctive norms (*b =* 4.47, *p =* 0.001), self-efficacy (*b =* 3.25, *p <* 0.001), and previous vaccination behavior (*b =* 45.33, *p <* 0.001) were significant predictors. For substantive interpretation of the effects, the Tobit coefficients have to be decomposed into the marginal effect (i.e., the impact of a predictor on the likelihood of vaccination intention reaching 100%) and the linear effect (i.e., the impact of a predictor on the likelihood of vaccination intention, given that it is below 100%). Table 3 presents the marginal effects and Table 4 presents the linear effects of each significant predictor of COVID-19 and influenza vaccination intention. The results were mixed for H4 in that descriptive norms had a negative impact on COVID-19 vaccination intention, which was opposite to the predicted direction. These results showed that H5 and H6 received support across both topics.

For RQ2, on the one hand, descriptive and injunctive norms had positive and similar effects (i.e., the effects were not significantly different from each other) on influenza vaccination intention. On the other hand, their effects were opposite in directions: descriptive norms had negative and injunctive norms had positive impacts on COVID−19 vaccination intention; however, the effects were not significantly different in terms of magnitude.

For RQ3, previous vaccination behavior, perceived virus severity, perceived vaccine effectiveness, attitude, injunctive norms, and self-efficacy positively predicted the increase in intention in percentages (or reduction in vaccine hesitancy) and the likelihood that the individual would become vaccine-inclined/-confident across both topics. Emotions (hope, happiness, and worry) reduced vaccine intention for influenza, but not for COVID-19. Perceived side effects severity negatively influenced COVID-19 vaccination intention, but not for influenza. Perceived side effects susceptibility and misinformation beliefs negatively impacted influenza vaccine intention, but not for COVID-19. For these factors, their impacts were probably mediated by attitude within each topic (see results for RQ1). That is, the factors that influenced COVID-19 and influenza vaccination intentions followed different patterns.

## 4. Discussion

Taking a behavioral approach to vaccine hesitancy (i.e., the reluctance or refusal to vaccinate despite the availability of vaccines), this study set out to investigate the predictors of vaccination intention for COVID-19 and influenza within the framework of the reasoned action approach [5] and the IBM model [6]. Given the similarities between COVID-19 and influenza, the fact that COVID-19 might require annual revaccination, and the possibility of a combination vaccine, it might be imperative that we investigate and compare the predictors of vaccination intention in terms of both the probability of vaccine uptake and vaccine confidence. Understanding and knowledge of the similarities and differences between predictors of COVID-19 and influenza vaccination intention could have significant implications and provide guidance for intervention strategy designs.

### 4.1. Strengths and Limitations

This study had several strengths. First, the two samples were nationally representative and independent of each other. Their sample sizes yielded sufficient statistical power for testing the predictors of vaccination intentions and for comparisons between COVID-19 and influenza. Second, recognizing the censored distributions in vaccination intentions, this study adopted the Tobit regression approach to properly estimate the impact of the predictors. Third, the Tobit analyses also allowed for more nuanced interpretations of the effects, namely, the predictors’ impact on the increase in vaccination likelihood (i.e., the linear component) and on the probability of vaccine uptake (i.e., the marginal component). The study also had some limitations. First of all, the samples were not probability or random samples, but quota samples. This might result in reduced external validity of the findings. Second, the data were essentially correlational in nature, which limits our ability to make causal inferences. Third, the behavioral approach to vaccine hesitancy assumes that 100% intention implies action. The literature on intention implementation [9] suggests that 100% intention does not necessarily materialize as actual action or goal achievement. Fourth, the study only examined the main effects of potential predictors but did not investigate any possible moderators. The results and conclusions from this study should be interpreted with its strengths and limitations in mind.

### 4.2. Comparing COVID-19 and Influenza

Data from two U.S. national samples (*N* = 1131 for COVID-19 and *N* = 1126 for influenza) collected from Qualtrics panels showed that the reasoned action approach and the IBM received support in that (1) attitude, social norms, and self-efficacy and (2) emotions and previous vaccination behavior significantly predicted vaccination intention. The differences between COVID-19 and influenza vaccines were as follows: (1) Attitude had a different set of affective predictors, (2) while the impact of emotions on vaccination intention was mediated by attitude for COVID-19, but not completely for influenza (RQ1). (3) Perceived susceptibility to negative consequences (from either the virus or vaccine side effects) and misinformation beliefs did not impact vaccination intention directly or indirectly via attitude, but perceived severity did, in the case of COVID-19. On the other hand, the impact of susceptibility (virus and vaccine side effects) and misinformation beliefs on vaccination intention was more salient and robust for influenza, and (4) the impact of descriptive norms on vaccination intention was negative for COVID-19, but positive for influenza. 

Examination of the distributions ruled out the possibility of ceiling effects for perceived severity and susceptibility variables, and for misinformation beliefs. Such differences in the pattern related to the affective and cognitive antecedents of attitude might be rooted in the depth and manner of information processing. Information processing might be more deliberate, systematic, and slower in the case of COVID-19, but more automatic, heuristic, and faster when it comes to influenza [57,77], which is probably due to the proximity, novelty, and salience of the risks and dangers posed by the SARS-CoV-2 virus and vaccines, relative to the influenza virus and vaccines. The different patterns regarding descriptive norms, on the other hand, might reflect the substance and biased nature of information processing. The observation of the majority behavior might be construed as an excuse for one not to get vaccinated (e.g., herd immunity might have been achieved because most people I know have received the vaccine; hence, it is okay for me not to get one), reflect the need for being esoteric and/or unique [78], or have triggered psychological reactance [79].

### 4.3. Implications for Interventions to Promote Vaccine Uptake and Confidence

The different patterns of affective predictors of vaccination intentions suggest that intervention messages should appeal to different emotions when promoting COVID-19 vs. influenza vaccines. For COVID-19, only negative emotions had effects on attitude, and not on vaccination intention directly. For influenza, both positive and negative emotions influenced attitude, as well as vaccination intention directly. Except for guilt and sadness, the impacts of the emotions were in line with their valence. This suggests that emotional appeals should focus on mitigating negative emotions when promoting COVID-19 vaccines, but enhancing positive emotions as well when encouraging influenza vaccination. The positive impact of guilt on attitude was consistent across the two topics. This suggests the utility of appeals to guilt by emphasizing the importance of herd immunity (i.e., if you do not get vaccinated, you are not contributing your fair share to the goal of achieving herd immunity) and by adopting the other-benefitting message frame [80].

The results concerning misinformation beliefs and the cognitive factors within the reasoned action approach also suggest messages should highlight different aspects of behavioral beliefs when promoting COVID-19 vs. influenza vaccines. On the one hand, campaign practitioners should seriously consider mitigating the impact of vaccine-related misinformation (e.g., through inoculation [81,82]) for both topics. On the other hand, while messages can address each of the reasoned action factors to facilitate influenza vaccination, not all of them would work for COVID-19. The results suggested that (1) highlighting susceptibility might not work well for COVID-19 messages since neither virus nor vaccine side effects susceptibility predicted vaccination intention, directly or indirectly; and (2) appeals to descriptive norms should probably be avoided due to the negative impact on vaccination intention. Injunctive norms should be highlighted instead when the normative influence approach is adopted.

It should be noted that such implications for message design are variable-based. Given that this study only investigated the main effects of potential predictors, providing guidance for audience segmentation and message tailoring/targeting was not possible based on these results. Given the number of significant predictors identified, traditional approaches to moderators based on two- or three-way interaction effects might not be very efficient in identifying segments of vaccine-hesitant individuals who can be targeted with similar message strategies, particularly not across the two topics, given the different patterns of predictors identified. An audience-centered, deep-level segmentation approach based on latent profile analysis might be better able to offer guidance for more precise, more proficient, and more cost-effective means of audience segmentation and message tailoring/targeting. Initial evidence using this approach [19,20,83] suggests that the profiles of vaccine-hesitant individuals overlap between COVID-19 and influenza, and that it could be feasible to target individuals with the same profiles using the same message strategies.

## Figures and Tables

**Table 1:** Demographic characteristics of the sample.

	COVID-19 (*N* = 1131)		Influenza (*N* = 1126)	
	*n*	%	*n*	%
Gender				
Male	491	43.4%	503	44.7%
Female	634	56.1%	614	54.5%
Non-binary	6	0.5%	9	0.8%
Race				
White	913	80.7%	918	81.5%
Black/African American	143	12.6%	130	11.5%
Hispanic/Latino	102	9%	98	8.7%
Asian or Pacific Islander	31	2.7%	57	5.0%
American Indian or Alaskan Native	35	3.1%	30	2.7%
Other/Prefer not to answer	9	0.8%	13	1.2%
Geographic area				
Urban	366	32.4%	392	34.8%
Suburban	455	40.2%	474	42.1%
Rural	310	27.4%	260	23.1%
Household annual income				
Less than USD 25,000	270	23.9%	239	21.2%
USD 25,000–49,999	332	29.4%	318	28.2%
USD 50,000–99,999	322	28.5%	345	30.6%
USD 100,000–149,999	132	11.7%	152	13.5%
USD 150,000 and above	75	6.6%	72	6.4%
Education				
Less than high school	39	3.4%	32	2.8%
High school diploma or equivalent	298	26.3%	358	31.8%
Some college, no degree	355	31.4%	334	29.7%
Associate degree	109	9.6%	77	6.8%
Bachelor’s degree	223	19.7%	220	19.5%
Master’s degree	91	8.0%	99	8.8%
Doctoral or professional degree	16	1.4%	6	0.5%
	*M*	*SD*	*M*	*SD*
Age	46.61	17.01	46.78	16.91

**Table 2:** Predicting attitude toward vaccines.

Predictors	COVID-19 (*N* = 1131)		Influenza (*N* = 1126)	
	∆R^2^	β	∆R^2^	β
Demographic variables ^1^	0.08 (*p* < 0.001)		0.07 (*p* < 0.001)	
Previous Vaccination Behavior	0.37 (*p <* 0.001)	0.16 (*p <* 0.001)	0.32 (*p <* 0.001)	0.18 (*p <* 0.001)
Political Orientation		−0.01 (*p =* 0.59)		−0.01 (*p =* 0.48)
Hope	0.10 (*p <* 0.001)	0.05 (*p =* 0.13)	0.12 (*p <* 0.001)	0.07 (*p =* 0.06)
Happiness		0.04 (*p =* 0.17)		0.09 (*p =* 0.02)
Sadness		0.02 (*p =* 0.50)		0.07 (*p =* 0.08)
Fear		0.05 (*p =* 0.17)		0.01 (*p =* 0.85)
Worry		−0.07 (*p =* 0.03)		−0.12 (*p =* 0.001)
Anger		−0.05 (*p =* 0.06)		−0.05 (*p =* 0.11)
Disgust		−0.05 (*p =* 0.06)		−0.07 (*p =* 0.02)
Guilt		0.09 (*p <* 0.001)		0.14 (*p <* 0.001)
Virus Severity	0.20 (*p <* 0.001)	0.10 (*p <* 0.001)	0.16 (*p <* 0.001)	0.07 (*p =* 0.001)
Virus Susceptibility		0.01 (*p =* 0.61)		0.01 (*p = 0*.69)
Vaccine Effectiveness		0.44 (*p <* 0.001)		0.33 (*p <* 0.001)
Side Effects Severity		−0.12 (*p <* 0.001)		−0.12 (*p <* 0.001)
Side Effects Susceptibility		0.01 (*p =* 0.74)		0.01 (*p =* 0.84)
Misinformation Beliefs		−0.12 (*p <* 0.001)		−0.17 (*p <* 0.001)

^1^ Age, gender (dummy variables), race (dummy variables), income, and education.

**Table 3:** Marginal effects ^1^ of predictors on vaccination intention ^2^.

Predictor	COVID-19				Influenza			
	$\frac{\Delta y}{\Delta x}$	*p*-Value	95% C.I.		$\frac{\Delta y}{\Delta x}$	*p*-Value	95% C.I.	
Previous Behavior	0.09	<0.001	0.07	0.11	0.31	<0.001	0.28	0.34
Virus Severity	0.03	<0.001	0.02	0.04	0.01	0.03	0.00	0.02
Virus Susceptibility	0.01	0.35	−0.01	0.02	0.02	0.005	0.01	0.03
Hope	0.00	0.83	−0.01	0.02	0.02	0.005	0.01	0.04
Happiness	0.01	0.09	−0.00	0.03	0.02	0.01	0.01	0.04
Worry	−0.01	0.43	−0.02	0.01	−0.02	0.01	−0.04	−0.01
Vaccine Effectiveness	0.04	<0.001	0.03	0.06	0.03	<0.001	0.02	0.04
Side Effects Severity	−0.03	<0.001	−0.04	−0.01	−0.01	0.15	−0.02	0.00
Side Effects Susceptibility	−0.00	0.90	−0.01	0.01	−0.02	0.002	−0.03	−0.01
Misinformation Beliefs	−0.01	0.17	−0.02	0.00	−0.03	0.002	−0.04	−0.01
Attitude	0.05	<0.001	0.04	0.07	0.04	<0.001	0.03	0.06
Descriptive Norms	−0.04	<0.001	−0.06	−0.02	0.03	0.006	0.01	0.05
Injunctive Norms	0.05	<0.001	0.03	0.07	0.03	0.001	0.01	0.05
Self-Efficacy	0.01	0.046	0.00	0.02	0.02	0.001	0.01	0.03

^1^ Marginal Effect = $\frac{\Delta y}{\Delta x}$, where ∆y = change in the probability that vaccination intention reaches the upper threshold of 100% and ∆x = change in a predictor. ^2^ Factors that did not have a direct effect on vaccination intention within either topic are not included. Coefficients in shaded color are non-significant.

**Table 4:** Linear effects ^1^ of predictors on vaccination intention ^2^.

Predictor	COVID-19				Influenza			
	$\frac{\Delta y}{\Delta x}$	*p*-Value	95% C.I.		$\frac{\Delta y}{\Delta x}$	*p*-Value	95% C.I.	
Previous Behavior	8.18	<0.001	6.50	9.86	29.45	<0.001	26.51	32.41
Virus Severity	2.78	<0.001	1.76	3.80	1.23	0.03	0.13	2.33
Virus Susceptibility	−0.47	0.35	−1.46	0.52	1.58	0.005	0.48	2.68
Hope	−0.14	0.83	−1.41	1.12	1.93	0.005	0.58	3.29
Happiness	1.10	0.09	−0.16	2.35	2.22	0.002	0.84	3.61
Worry	−0.55	0.43	−1.93	0.82	−1.97	0.01	−3.49	−0.46
Vaccine Effectiveness	4.13	<0.001	30.01	5.25	2.80	<0.001	1.66	3.93
Side Effects Severity	−2.40	<0.001	−3.61	−1.18	0.95	0.15	−0.35	2.26
Side Effects Susceptibility	0.07	0.90	−0.99	1.13	−1.88	0.002	−3.07	−0.69
Misinformation Beliefs	−0.90	0.17	−2.18	0.37	−2.37	0.002	−3.89	−0.85
Attitude	4.84	<0.001	3.59	6.10	4.04	<0.001	2.78	5.31
Descriptive Norms	−3.55	0.001	−5.47	−1.63	2.48	0.007	0.69	4.26
Injunctive Norms	4.28	<0.001	2.54	6.02	2.90	0.001	1.18	4.64
Self-Efficacy	1.13	0.046	0.02	2.23	2.11	<0.001	0.98	3.24

^1^ Linear Effect = $\frac{\Delta y}{\Delta x}$, where ∆y = change in vaccination intention (in percentage) given that it remains below the upper threshold of 100% and ∆x = change in a predictor. ^2^ Factors that did not have a direct effect on vaccination intention within either topic are not included. Coefficients in the shaded color are non-significant.

## Data Availability

The data files used in this study are available at: https://osf.io/4y3h6/?view_only=c89dfbff34c84731bcbaf932fa726b51.

## References

[1] Galagali P.M., Kinikar A.A., Kumar V.S. (2022). Vaccine hesitancy: Obstacles and challenges. Curr. Pediatr. Rep..

[2] Suryawanshi Y.N., Biswas D.A., Biswas D. (2023). Herd Immunity to fight against COVID-19: A narrative review. Cureus.

[3] Dhanani L.Y., Franz B. (2022). A meta-analysis of COVID-19 vaccine attitudes and demographic characteristics in the United States. Public Health.

[4] Grochowska M., Ratajczak A., Zdunek G., Adamiec A., Waszkiewicz P., Feleszko W. (2021). A comparison of the level of acceptance and hesitancy towards the influenza vaccine and the forthcoming COVID-19 vaccine in the medical community. Vaccines.

[5] Fishbein M., Ajzen I. (2010). Predicting and Changing Behavior: The Reasoned Action Approach.

[6] Montaño D.E., Kasprzyk D., Glanz K., Rimer B.K., Viswanath K. (2015). Theory of reasoned action, theory of planned behavior, and the integrated behavioral model. Health Behavior and Health Education: Theory, Research, and Practice.

[7] Bussink-Voorend D., Hautvast J.L., Vandeberg L., Visser O., Hulscher M.E. (2022). A systematic literature review to clarify the concept of vaccine hesitancy. Nat. Hum. Behav..

[8] MacDonald N.E. (2015). Vaccine hesitancy: Definition, scope and determinants. Vaccine.

[9] Gollwitzer P.M., Sheeran P. (2006). Implementation intentions and goal achievement: A meta-analysis of effects and processes. Adv. Exp. Soc. Psychol..

[10] Dubé E., Laberge C., Guay M., Bramadat P., Roy R., Bettinger J.A. (2013). Vaccine hesitancy: An overview. Hum. Vaccines Immunother..

[11] Salmon D.A., Dudley M.Z., Glanz J.M., Omer S.B. (2015). Vaccine hesitancy: Causes, consequences, and a call to action. Vaccine.

[12] Albarracin D., Johnson B.T., Fishbein M., Muellerleile P.A. (2001). Theories of reasoned action and planned behaviors as models of condom use: A meta-analysis. Psychol. Bull..

[13] Floyd D.L., Prentice-Dunn S., Rogers R.W. (2000). A meta-analysis of research on protection motivation theory. J. Appl. Soc. Psychol..

[14] McEachan R., Taylor N., Harrison R., Lawton R., Gardner P., Conner M. (2016). Meta-analysis of the reasoned action approach (RAA) to understanding health behaviors. Ann. Behav. Med..

[15] Kim M.S., Hunter J.E. (1993). Relationships among attitudes, behavioral intentions, and behavior: A meta-analysis of past research: II. Commun. Res..

[16] Chou W.S., Budenz A. (2020). Considering emotions in COVID-19 vaccine communication: Addressing vaccine hesitancy and fostering vaccine confidence. Health Commun..

[17] Tomljenovic H., Bubic A., Erceg N. (2020). It just doesn’t feel right–the relevance of emotions and intuition for parental vaccine conspiracy beliefs and vaccination uptake. Psychol. Health.

[18] Gavaruzzi T., Caserotti M., Leo I., Tasso A., Speri L., Ferro A., Fretti E., Sannino A., Rubaltelli E., Lotto L. (2021). The role of emotional competencies in parents’ vaccine hesitancy. Vaccines.

[19] Zhou Y., Li R., Shen L. (2023). Targeting COVID-19 vaccine-hesitancy in college students: An audience-centered approach. J. Am. Coll. Health.

[20] Zhou Y., Li R., Shen L. (2023). Psychological profiles of COVID vaccine-hesitant individuals and implications for vaccine message design strategies. Vaccine.

[21] Russell P.S., Frackowiak M., Cohen-Chen S., Rusconi P., Fasoli F. (2023). Induced gratitude and hope, and experienced fear, but not experienced disgust, facilitate COVID-19 prevention. Cogn. Emot..

[22] Dillard J.P., Peck E. (2001). Persuasion and the structure of affect: Dual systems and discrete emotions as complementary models. Hum. Commun. Res..

[23] Russell J.A., Carroll J.M. (1999). On the bipolarity of positive and negative affect. Psychol. Bull..

[24] Bohner G., Crow K., Erb H., Schwartz N. (1992). Affect and persuasion: Mood effects on the processing of message content and context cues and on subsequent behavior. Eur. J. Soc. Psychol..

[25] Schwarz N., Higgins E.T., Sorrentino R.M. (1990). Feeling as information: Information and motivational function of affective states. Handbook of Motivation and Cognition.

[26] Öhman A., Lewis M., Haviland-Jones J.M., Barrett L.F. (2008). Fear and anxiety: Overlaps and dissociations. Handbook of Emotions.

[27] Scherer K.R., Schorr A., Johnstone T. (2001). Appraisal Processes in Emotion: Theory, Methods, Research.

[28] Lazarus R.S. (1991). Emotion and Adaptation.

[29] Rogers R.W., Prentice-Dunn S., Gochman D.S. (1997). Protection motivation theory. Handbook of Health Behavior Research 1: Personal and Social Determinants.

[30] Joshi A., Kaur M., Kaur R., Grover A., Nash D., El-Mohandes A. (2021). Predictors of COVID-19 vaccine acceptance, intention, and hesitancy: A scoping review. Front. Public Health.

[31] Sheeran P., Maki A., Montanaro E., Avishai-Yitshak A., Bryan A., Klein W.M.P., Miles E., Rothman A.J. (2016). The impact of changing attitudes, norms, and self-efficacy on health-related intentions and behavior: A meta-analysis. Health Psychol..

[32] Zhou Y., Shen L. (2023). Confirmation bias and the persistence of misinformation on climate change. Commun. Res..

[33] McKee C., Bohannon K. (2016). Exploring the reasons behind parental refusal of vaccines. J. Pediatr. Pharmacol. Ther..

[34] Kouzy R., Jaoude J.A., Kraitem A., El Alam M.B., Karam B., Adib E., Zarka J., Traboulsi C., Akl E., Baddour K. (2020). Coronavirus goes viral: Quantifying the COVID-19 misinformation epidemic on Twitter. Cureus.

[35] Allington D., Duffy B., Wessely S., Dhavan N., Rubin J. (2021). Health-protective behaviour, social media usage and conspiracy belief during the COVID-19 public health emergency. Psychol. Med..

[36] Hall Jamieson K., Albarracín D. (2020). The relation between media consumption and misinformation at the outset of the SARS-CoV-2 pandemic in the US. Harv. Kennedy Sch. Misinformation Rev..

[37] Jennings W., Stoker G., Bunting H., Valgarðsson V.O., Gaskell J., Devine D., McKay L., Mills M.C. (2021). Lack of trust, conspiracy beliefs, and social media use predict COVID-19 vaccine hesitancy. Vaccines.

[38] Neely S.R., Eldredge C., Ersing R., Remington C. (2022). Vaccine hesitancy and exposure to misinformation: A survey analysis. J. Gen. Intern. Med..

[39] Rasul M.E., Ahmed S. (2023). Not all conservatives are vaccine hesitant: Examining the influence of misinformation exposure, political ideology, and flu vaccine acceptance on COVID-19 vaccine hesitancy. Vaccines.

[40] Zimmerman T., Shiroma K., Fleischmann K., Xie B., Jia C., Verma N., Lee M.K. (2022). Misinformation and COVID-19 vaccine hesitancy. Vaccine.

[41] Skinner C.S., Tiro J., Champion V.L., Glanz K., Rimer B.K., Viswanath K. (2015). The health belief model. Health Behavior: Theory, Research, and Practice.

[42] Rimal R.N., Real K. (2005). How behaviors are influenced by perceived norms: A test of the theory of normative social behavior. Commun. Res..

[43] Kruglanski A.W., Pierro A., Mannetti L., De Grada E. (2006). Groups as epistemic providers: Need for closure and the unfolding of group-centrism. Psychol. Rev..

[44] Lapinski M.K., Rimal R.N. (2005). An explication of social norms. Commun. Theory.

[45] Chung A., Rimal R.N. (2016). Social norms: A review. Rev. Commun. Res..

[46] Gavrilets S. (2020). The dynamics of injunctive social norms. Evol. Hum. Sci..

[47] Rhodes N., Shulman H.C., McClaran N. (2020). Changing norms: A meta-analytic integration of research on social norms appeals. Hum. Commun. Res..

[48] Miller D.T., Prentice D.A. (2016). Changing norms to change behavior. Annu. Rev. Psychol..

[49] Reynolds K.J. (2019). Social norms and how they impact behavior. Nat. Hum. Behav..

[50] Akfırat S., Bayrak F., Üzümçeker E., Ergiyen T., Yurtbakan T., Uysal M.S. (2023). The roles of social norms and leadership in health communication in the context of COVID-19. Soc. Sci. Med..

[51] Jaffe A.E., Graupensperger S., Blayney J.A., Duckworth J.C., Stappenbeck C.A. (2022). The role of perceived social norms in college student vaccine hesitancy: Implications for COVID-19 prevention strategies. Vaccine.

[52] Sinclair S., Agerström J. (2023). Do social norms influence young people’s willingness to take the COVID-19 vaccine?. Health Commun..

[53] Monto A.S. (2021). The future of SARS-CoV-2 vaccination—Lessons from influenza. N. Engl. J. Med..

[54] Bandura A. (2006). Toward a psychology of human agency. Perspect. Psychol. Sci..

[55] Cialdini R.B. (2021). Influence, New and Expanded: The Psychology of Persuasion.

[56] Dillard J.P. (1993). Persuasion past and present: Attitudes aren’t what they used to be. Commun. Monogr..

[57] Eagly A.H., Chaiken S. (1993). The Psychology of Attitudes.

[58] Albarracin D. (2021). Action and Inaction in a Social World: Predicting and Changing Attitudes and Behavior.

[59] Albarracin D., Wyer R.S. (2000). The cognitive impact of past behavior: Influences on beliefs, attitudes, and future behavioral decision. J. Personal. Soc. Psychol..

[60] Kidwell B., Jewell R.D. (2008). The influence of past behavior on behavioral intent: An information-processing explanation. Psychol. Mark..

[61] Hagger M.S., Hamilton K., Phipps D.J., Protogerou C., Zhang C.-Q., Girelli L., Mallia L., Lucidi F. (2023). Effects of habit and intention on behavior: Meta-analysis and test of key moderators. Motiv. Sci..

[62] Dillard J.P., Shen L. (2018). Threat appeals as multi-emotion messages: An argument structure model of fear and disgust. Hum. Commun. Res..

[63] Bollen K., Diamantopoulos A. (2017). In defense of causal-formative indicators: A minority report. Psychol. Methods.

[64] Townsend J.P., Hassler H., Dornburg A. (2023). Infection by SARS-CoV-2 with alternate frequencies of mRNA vaccine boosting. Med. Virol..

[65] Bolsen T., Palm R. (2021). Politicization and COVID-19 vaccine resistance in the U.S. Prog. Mol. Biol. Transl. Sci..

[66] Cao J., Ramirez C.M., Alvarez R.M. (2023). The politics of vaccine hesitancy in the United States. Soc. Sci. Q..

[67] Sabahelzain M.M., Hartigan-Go K., Larson H.J. (2021). The politics of COVID-19 vaccine confidence. Curr. Opin. Immunol..

[68] Cohen A.C. (1991). Truncated and Censored Samples. Theory and Applications.

[69] Long S. (1997). Regression Models for Categorical and Limited Dependent Variables.

[70] Winship C., Morgan S.L. (1999). The estimation of causal effects from observational data. Annu. Rev. Sociol..

[71] McDonald J.F., Moffitt R.A. (1980). The uses of tobit analysis. Rev. Econ. Stat..

[72] Tobin J. (1958). Estimation of relationships for limited dependent variables. Econometrica.

[73] Pan Z., Shen L., Paek H., Sun Y. (2006). Mobilizing political talk in a presidential campaign: An examination of campaign effects in a deliberative framework. Commun. Res..

[74] Shen L., Bigsby E. (2010). Behavioral activation/inhibition systems and emotions: A test of valence vs. action tendency hypotheses. Commun. Monogr..

[75] Prochaska J.O., Redding C.A., Eves K.E., Glanz K., Rimer B.K., Viswanath K. (2015). The transtheoretical model and stages of change. Health Behavior: Theory, Research, and Practice.

[76] Faul F., Erdfelder E., Lang A.-G., Buchner A. (2007). G*Power 3: A flexible statistical power analysis program for the social, behavioral, and biomedical sciences. Behav. Res. Methods.

[77] Kahneman D. (2013). Thinking, Fast and Low.

[78] Bailey A. (1942). Esoteric Psychology.

[79] Dillard J.P., Tian X., Cruz S.M., Smith R.A., Shen L. (2023). Persuasive messages, social norms, and reactance: A study of masking behavior during a COVID-19 campus health campaign. Health Commun..

[80] Kelly B.J., Hornik R.C. (2016). Effects of framing health messages in terms of benefits to loved ones or others: An experimental study. Health Commun..

[81] Boman C. (2023). Protecting against disinformation: Using inoculation to cultivate reactance toward astroturf attacks. J. Public Relat. Res..

[82] Van der Linden S., Roozenbeek J., Compton J. (2020). Inoculating against fake news about COVID-19. Front. Psychol..

[83] Shen L. Toward deep-level person-centered message tailoring to address COVID-19 and influenza vaccine-hesitancy: A latent profile analysis approach. Proceedings of the National Communication Association Conference.

